# Effects of phytocompound Precocene 1 on the expression and functionality of the P450 gene in λ-cyhalothrin-resistant *Spodoptera litura* (Fab.)

**DOI:** 10.3389/fphys.2022.900570

**Published:** 2022-11-10

**Authors:** Narayanan Shyam-Sundar, Ramakrishnan Ramasubramanian, Sengodan Karthi, Sengottayan Senthil-Nathan, Kanagaraj Muthu-Pandian Chanthini, Haridoss Sivanesh, Vethamonickam Stanley-Raja, Govindaraju Ramkumar, Kilapavoor Raman Narayanan, Shahid Mahboob, Khalid Abdullah Al-Ghanim, Ahmed Abdel-Megeed, Patcharin Krutmuang

**Affiliations:** ^1^ Division of Biopesticides and Environmental Toxicology, Sri Paramakalyani Centre for Excellence in Environmental Sciences, Manonmaniam Sundaranar University, Tirunelveli, Tamil Nadu, India; ^2^ Department of Entomology, University of Kentucky, Lexington, KY, United States; ^3^ Department of Zoology, Sri Paramakalyani College, Tirunelveli, Tamil Nadu, India; ^4^ Department of Zoology, College of Science, King Saud University, Riyadh, Saudi Arabia; ^5^ Department of Plant Protection, Faculty of Agriculture Saba Basha, Alexandria University, Alexandria, Egypt; ^6^ Department of Entomology and Plant Pathology, Faculty of Agriculture, Chiang Mai University, Chiang Mai, Thailand; ^7^ Innovative Agriculture Research Center, Faculty of Agriculture, Chiang Mai University, Chiang Mai, Thailand

**Keywords:** phytochemicals, Precocene 1, pyrethroid, growth regulator, anti-juvenile hormone, cytochrome P450, qRT-PCR, gene expression

## Abstract

*Spodoptera litura* (Fabricius) is an agriculturally significant polyphagous insect pest that has evolved a high level of resistance to conventional insecticides. A dietary assay was used in this work to assess the resilience of field populations of *S. litura* to λ-cyhalothrin. Analysis of the function and expression of the cytochrome P450 gene was used to test the sensitivity of *S. litura* larvae to sub-lethal concentrations of the insecticidal plant chemical Precocene 1, both by itself and in combination with λ-cyhalothrin. The activity of esterase enzymes (α and β) was found to decrease 48 h post treatment with Precocene 1. The activity of GST enzyme and cytochrome P450 increased with Precocene 1 treatment post 48 h, however. Expression studies revealed the modulation by Precocene 1 of cytochrome P450 genes, *CYP4M16*, *CYP4M15*, *CYP4S8V4*, *CYP4G31*, and *CYP4L10*. While *CYP4M16* expression was stimulated the most by the synergistic Precocene 1 + λ–cyhalothrin treatment, expression of *CYP4G31* was the most down-regulated by Precocene 1 exposure. Hence, it is evident that λ–cyhalothrin-resistant pest populations are still sensitive to Precocene 1 at a sublethal concentration that is nevertheless capable of hindering their development. Precocene 1 can therefore be considered a potent candidate for the effective management of insecticide-resilient *S. litura*.

## Introduction

*Spodoptera litura* (Fabricius) (Lepidoptera: Noctuidae) is a cosmopolitan insect pest, widely distributed throughout tropical and subtropical regions (Senthil-Nathan and Kalaivani 2005,^2006^; Senthil-Nathan, 2015) due to its high levels of reproduction, detoxification mechanisms, and migratory patterns (Liu et al., 2010; Amala et al., 2021). These insects infect more than 40 plant families, including 180 host plants. The phytophagous pest resists insecticide attack through various mechanisms, such as by lowering the quantity of allelochemicals, de-activating xenobiotics metabolically (Peng et al., 2017), skillfully avoiding the defense-induced area of the leaf surface of plants (Senthil-Nathan, 2019), developing insensitivity in plants toward protease inhibitors (Senthil-Nathan, 2019), and by intensively eliminating allelochemicals through ATP-binding cassette transporters (ABC transporters) (Meena et al., 2022).

Pest control practices via indiscriminate application of synthetic insecticides lead to resistance development and hence the failure of the control measure (Murali-Baskaran et al., 2021; Subaharan et al., 2021). Resistance to insecticides in lepidopteran pests is mainly by detoxification and target site insensitivity, involving enzymes such as esterase, glutathione complex, and cytochrome P450 (Tang et al., 2022). Organophosphate (OP) and carbamate insecticides inhibit the activity of AChE in insects through the process of phosphorylating or carbamylating the serine residues at the target site (Cao et al., 2020). Resistance to organophosphates and carbamates in *Helicoverpa armigera* and *S. litura* has already been reported (Selin-Rani et al., 2016). The epsilon class GSTs of *S. litura* are good enough to detoxify DDT (dichloro-diphenyl-trichloroethane) and deltamethrin (Deng et al., 2009). Excessive application of cypermethrin and pyrethroid develops resistance in *S. litura* (Shyam-Sundar et al., 2021a, 2021b) by way of decreased penetration, target site sensitivity alteration, and enhancement of detoxification enzyme activity, such as that of cytochrome P450 monooxygenase (P450), carboxylesterase (CarE), and glutathione S-transferase (GST) (Ahmad and McCaffery, 1999; Dong et al., 2016). Esterase exhibits a broad spectrum of accuracy, capable of cleaving tri-ester-phosphates, halides, esters, thioesters, amides, and peptides. The modality of esterases in detoxifying insecticides is well reported (Saleem and Shakoori, 1996). These enzymes are synthesized in the biochemical pathway during the developmental stages of the larva (Huang and Han, 2007). Increase in esterase activity when exposed to synthetic pesticide is one of the main resistance mechanisms in pests (Kranthi et al., 2002). Glutathione-S-transferase (GST) belongs to a protein family, and plays a major role in the detoxification of xenobiotics by converting them into less toxic water-soluble products (Singh et al., 2001). GST develops resistance against organophosphorus and pyrethroid insecticides in a wide range of insect pests (Senthil-Nathan, 2020). Insecticide-resistant strains of various insects reveal a correlation, in terms of an upsurge in expression of gene and GST activity, as part of their insecticide resistance (Li et al., 2007).

All organisms contain the cytochrome P450 monooxygenase (P450 or CYP) family, with a varied functional group of hemoproteins (Li W. et al., 2004; Guo et al., 2011; Nelson, 2011; Cheng et al., 2017). In insects, the role of P450 enzymes involves growth, development, biosynthesis, the regulation of hormones, and the metabolism of the xenobiotics (Nelson, 2011; Pottier et al., 2012) and insecticides that create resistance (Zhou et al., 2010; Guo et al., 2011; Sparks et al., 2021). Insect P450 genes consist of CYP2, CYP3, and CYP4 and the mitochondrial CYP clades (Feyereisen, 2011; Schuler and Berenbaum, 2013). Moreover, Clade 3 has the CYP6 and CYP9 families (Li et al., 2007), and particularly the CYP6 family, which are connected to the biochemical pathways that break down the derivatives of plant allelochemicals and lead to changes in feeding behavior and growth patterns (Li X. C. et al., 2004; Despre et al., 2007; Zeng et al., 2007; Niu et al., 2011; Dawkar et al., 2013).

The mechanism of the detoxifying P450 enzyme in insects helps them resist phytoconstituents and chemical insecticides (Sasabe et al., 2004; Mao et al., 2006; Despre et al., 2007; Bautista et al., 2009; Niu et al., 2011; Pentzold et al., 2014; Lu et al., 2021). Various P450s are capable of metabolizing a single substrate, and a single P450 is able to metabolize multiple substrates (Meunier et al., 2004). Phytochemical-inducible P450s are required for the development of cross-tolerance in insecticides (Li et al., 2000). The overexpression of P450s results in increased insecticide resistance, and tolerance to allelochemicals has been reported in various orders of insects, such as Lepidoptera, Diptera, Coleoptera, Hemiptera, and Hymenoptera (Bass et al., 2011; Johnson et al., 2012; Liang et al., 2015; Chen C. et al., 2017; Chen Y. et al., 2017; Wang R. L. et al., 2018; Wang X. et al., 2018; Hass et al., 2022). The role of cytochrome P450 genes and enzymes in *S. litura* for detoxifying host plant allelochemicals and other xenobiotics has not been much explored.

The major damage to agricultural crops results mainly from attack by lepidopteran insects (Shu, 2012; Selin-Rani et al., 2016). *S. litura*, in particular, develops resistance when exposed to synthetic pesticides (Jahan et al., 2008; Shyam-Sundar et al., 2021a, 2021b). The present study is aimed at finding the strategy of using allelochemicals for developing resistance when exposed to insecticides. In light of the previously mentioned, the present study scrutinizes the impact of Precocene 1, and its influence on detoxifying enzyme activity, and on the expression levels of five P450 genes in *S. litura* larvae (*CYP4M16*, *CYP4M15*, *CYP4S8V4*, *CYP4G31*, and *CYP4L10*)*,* upon exposure to λ-cyhalothrin.

## Materials and methods

### Insects

The larvae of *S. litura* were obtained from agricultural land in Kadayam (latitude 8.8213° N, longitude 77.3741° E), Tenkasi District, India, and cultured in the Biopesticides and Ecotoxicology Laboratory (BET Lab), SPKCEES, Manonmaniam Sundaranar University, Alwarkurichi. To maintain generation, the larvae were kept in an insectary at 27 ± 1°C, with relative humidity (RD) of 85%, under a 12:12 L:D photoperiod schedule. Castor leaves were given as feed, and the adults that emerged were placed in a container (10 × 10 × 7 cm), with castor leaves for pairing (1 male: 2 females). A 10% honey solution was given to adults for oviposition, and a sanitary black cloth was used to cover the cage.

### Chemicals

Precocene 1, phenyl methylsulfonyl fluoride (PMSF), and NADPH were purchased from Sigma-Aldrich (Mumbai, India). The λ-cyhalothrin was purchased commercially from a local chemical company in India. Dithiothreitol (DTT), glycerol, and Tris were obtained from Himedia Chemicals, Hyderabad, India, while 7-ethoxycoumarin, 7-hydroxycoumarin, EDTA, and bovine serum albumin were obtained from Sigma-Aldrich, Mumbai, India. Reagent grade chemicals and solvents were used.

### Preparation of chemically supplemented diets

Preparation of an artificial and chemically supplemented diet followed previously published protocols (Karthi and Shivakumar, 2015; Chen C. et al., 2017). The Precocene 1 was dissolved in 1% dimethyl sulfoxide (DMSO). For the control, the artificial feed was made with the addition of 1% of DMSO. Distilled water containing 0.1% (v/v) Triton X-100 and 1% DMSO was used to dilute the λ-cyhalothrin insecticide, plus 1% DMSO, and treated as stock solution. For bioassays, 15 ml of stock solution was pipetted and sterilized in plastic cups of 4.0 cm in diameter × 3.0 cm in height. Agar was added and stirred into the liquid artificial diet for 2 min, which was then allowed to solidify at 40–45°C. A similar protocol was carried out for the preparation of the control diet, but without adding insecticides, and the control was then maintained at 4°C prior to use.

### Toxicity bioassay

Third-instar larvae were used to find the effects of Precocene 1 uptake and tolerance to λ-cyhalothrin for all instances, as this developmental stage allows for observation of weight gain and mortality. 0.2% Precocene 1 was added to the artificial diet for 48 h before bioassay, and the control larvae were given the artificial diet containing the 0.1% DMSO solution. In total, twenty-five third-instar larvae were used for the experiment with five replications. In addition, in order to study the toxicity of λ-cyhalothrin, a diet merger methodology was followed using third-instar larvae (Karthi and Shivakumar, 2015). A standard solution (2,500 mg/L) of λ-cyhalothrin was dissolved in deionized water, having 0.1% (v/v) Triton X-100 and 1% DMSO for bioassays at concentrations of 150, 180, 210, 240, and 270 mg/L. A known volume of insecticide from the abovementioned solutions was added to tiny 20-ml sterilized plastic cups (4.0 cm × 3.0 cm), into which the artificial liquid diet was incorporated and stirred for 2 min. For the control, the same quantity of 0.1% (v/v) Triton X-100 and 1% DMSO was included in the diet and the cups were covered with perforated lids for the purpose of ventilation. For every bioassay series, and for each insecticide concentration, twenty-five larvae from the pretreated 0.2% Precocene 1 group, and from the non-exposed group, were used. The diet without chemical treatment was given to the control larvae. Mortality was recorded at 72 h post-treatment, and five replications were carried out in each experimental study.

### Synergistic impact of PBO on the toxic nature of insecticide

Piperonyl butoxide (PBO) was used as the synergist, and its presence or absence was evaluated as described earlier. *S. litura* larvae were fed with an artificial diet with or without 0.2% Precocene 1 for 48 h. To obtain the concentration of 25 mg/L, the PBO was dissolved in acetone. Using a micro-syringe, 10 µg of PBO/larva was applied topically in the dorsal prothorax region of individual larvae of *S. litura.* After sterilization, plastic cups were used to place the 2 h PBO-exposed larvae of *S. litura*, to which were added different concentrations of insecticide solutions, namely, 150, 180, 210, 240, and 270 of λ-cyhalothrin mg/L, with or without 0.2% Precocene 1, to assess the toxicity of λ**-**cyhalothrin. The diet with Precocene 1 was fed to the larvae for 48 h, without the pre-treated PBO of λ-cyhalothrin, and the larvae were kept in cups with perforated lids. Every bioassay was conducted in triplicate.

### Whole-body homogenate preparation for enzyme assay

Ten fourth-instar larvae were treated with Precocene 1 and then homogenized on ice with 0.1 M phosphate buffer (7.2 pH), containing 1 mM EDTA, 1 mM DTT, 1 mM PTU, 1 mM PMSF, and 20% glycerol. Tissues were collected after 24 and 48 h periods of homogenization in 2 ml of the buffer, and centrifugation was performed at 4°C, 10,000 g for 15 min. Solid debris and cellular materials were separated and the supernatant was transferred into Eppendorf tubes. These were placed on ice for the assay of carboxyl esterase, glutathione-S-transferase, and cytochrome P450. The total content of protein was then estimated using the procedure of Lowry et al. (1951).

### Carboxyl esterase assays

The α and β-carboxylesterase activity was determined (Kranthi, 2005) in the larval extracts prepared with phosphate potassium buffer (0.1 M: pH 7.2), and with 20 μl; 84 μg protein. The extract was added to 500 μl buffer (0.3 mMα- or β-naphthyl acetate in 0.1 M phosphate potassium at pH 7.2, containing 1% acetone), and followed by incubation at 30°C for 20 min. To this, 0.3 and 3.3 percentages of Fast Blue B and sodium dodecyl sulfate (SDS) were added, respectively. After centrifugation (3,000x g, 28°C), the supernatant was collected and absorbance was recorded at 590 nm. One unit of enzyme activity determines the quantity of enzyme required to generate 1 μmol of α- or β-naphthol per minute.

### Glutathione-S-transferase activity

Glutathione S-transferase assays were carried out, following the protocol of Kao et al. (1989), with reduced glutathione GSH as the substrate with 1-chloro 2,4-dinitrobenzene. Fourth-instar larvae were homogenized with 250 μl of sodium phosphate buffer (50 mM: pH 7.2) and centrifuged at 15,000×g at 4°C for 20 min. The union of the thiol group of glutathione with the substrate comprising 1-chloro-2, 4-dinitrobenzene (CDNB) was estimated using the Sigma-Aldrich (Catalog 0410, Bangalore) GST assay kit. Each well was loaded with 20 μl of the homogenate, along with 200 μl of Dulbecco’s phosphate buffer (Sigma-Aldrich, Bangalore, IN), reduced glutathione (4 mM), and CDNB (2 mM). The GST activity was conjugated as a μmol/mg protein/min substrate.

### Cytochrome P450 activity

Determination of Cyt P450 activity was carried out by peroxidation of a TMBZ assay following the protocol of Brogdon et al. (1989) with minimal alteration. After adding 250 μl of 0.05 M potassium phosphate buffer (pH 7.0) to 50 μl microfuge supernatant and 500 μl TMBZ solution (0.05% 3,3′,5,5′ tetramethyl benzidine, i.e., TMBZ +5 ml methanol +15 ml sodium acetate buffer 0.25 M pH 5.0), the mixture was prepared by adding 200 μl of 3% hydrogen peroxide and incubated at room temperature for 30 min. The reading was taken at 630 nm absorbance and calculated by comparison with the standard curve of cytochrome C.

### Extraction of total RNA and synthesis of cDNA

The total content of the RNA in *S. litura* larvae was extracted by TRIzol (Invitrogen). Synthesis of cDNA was performed in 20 µl of reactant, having 1 µl of total RNA, 0.6 µl of forward primer (10 pmol), 0.6 µl of reverse primer (10 pmol), and 20 units of RNase inhibitor, 1 µl of dNTP mixture (10 mM each), and 1 µl oligo (dT)18 primer (50 µM) at 42°C , for 1 h by reverse transcription. The concentration of RNA was quantified at 260–280 nm absorbance. The quality was assessed by agarose gel electrophoresis, and staining was performed with ethidium bromide (EB).

### Analysis of real-time RT-PCR

To estimate the amount of RNA using agarose gel electrophoresis, the reverse transcription to cDNA from 1 µg of total RNA was conducted with a PrimeScript^®^ RT Reagent Kit with gDNA Eraser (Perfect Real Time) (TaKaRa, Japan) according to the manufacturer’s protocol. To study the RT-PCR, primers (Table 1) were planned using Primer 3 software (Applied Biosystems). SYBR Green qPCR was carried out in a 0.2 ml PCR 8-tube strip with flat 8-cap strips (Axygen, USA), employing an iQ5 real-time PCR detection system (Bio-Rad, United States). The prepared 20 µl PCR reaction consisted of 2 µl cDNA template, 10 µl 2 X SYBR^®^ Premix Ex Taq™ II (Perfect Real Time) (TaKaRa, Japan), 0.8 µl of each primer (10 pmol/µl), and 6.4 µl ddH_2_O. The RT-PCR program was carried out using the melting curve dissociation methodology (from 55°C to 95°C), and by following the thermal conditions: initial denaturation 95°C for 30 s, subsequently 40 cycles of 95°C for 5 s, and 60°C for 30 s. A melting curve analysis was then performed to assess the specificity and consistency of the PCR products. The expression levels of target genes were calculated with the 2^−ΔΔCT^ method and normalized to the internal housekeeping gene β-actin.

**TABLE 1:** Primers used in this study for quantitative real time polymerase chain reaction (qRT-PCR).

S. No	Primer name	Primer sequence (5′-3′)	Size (bp)	NCBI accession numbers
1	CYP4M16	GGC​GAA​CGA​ACC​TGA​AAT​A	103	DQ355382
		CTT​CAT​CTG​ACT​CAA​GTC​TTC​C		
2	CYP4M15	CCC​ACC​AGT​GCA​CTT​TAT​TA	104	DQ352139
		GCA​GGT​CTA​AGA​TCA​GAA​TGT		
3	CYP4S8v4	AGT​ATT​TGG​AGG​CAG​TCA​TC	116	DQ355381
		CGT​ACC​CTT​CTT​CAC​TGT​TAT		
4	CYP4G31	CAC​CCT​GCA​GAT​GAA​GTA​TT	119	DQ350813
		CGT​AGT​TGT​TGG​TAG​CGA​TT		
5	CYP4L10	TTG​AGC​GAA​GGA​TAA​CAA​GAG	99	DQ352134
		CTGGTTGGCGTTGAATCT		
6	β-actin	TGA​GAC​ATT​CAA​CAC​CCC​TG	132	KJ612090.1
		CCT​TCA​TAG​ATT​GGG​ACA​GTG		

### Statistical analysis

Data are represented in mean ± SD. Resulting pairs were compared using the Student’s t-test. One-way ANOVA was applied to determine the significant differences (*p* < 0.05) for different groups by using the Tukey test. Statistical analysis was carried out with SPSS 11.5 software.

## Results

### λ-cyhalothrin toxicity to *S. litura* larvae and synergist activity

The impact of Precocene 1 and its synergistic effect with PBO against the tolerance and sensitivity of *S. litura* to λ**-**cyhalothrin is presented in Table 2. The LC_50_ value of larvae of *S. litura* treated with PBO was 91.89 mg/L. The effect of Precocene 1 alone, tested for LC_50_ in the larvae of *S. litura*, was found to be lower (78.05 mg ai/L), a considerable decrease due to the synergistic effect of PBO with Precocene 1 (61.06 mg ai/L).

**TABLE 2:** Synergistic effect of piperonyl butoxide on the susceptibility of third-instar larvae of *S. litura* to lambda-cyhalothrin after the ingestion of Precocene 1.

Treatment	LC_50_ (mg a.i./L)	95% CL	Slope ±SE	RR	χ^2^
Piperonyl butoxide (PBO)	91.89	81.67 ± 99.08	3.154 ± 0.39	7.5	2.157
Precocene 1	78.05	71.08 ± 86.05	2.871 ± 0.67	6.37	1.763
Precocene 1 + piperonyl butoxide (PBO)	61.06	55.04 ± 68.87	2.172 ± 0.05	4.98	1.235
Susceptible	12.24	11.85 ± 13.31	1.021 ± 0.03	—	0.135

Here, LC_50_ = lethal concentration to kill 50% of the population; a. i. = active ingredient; CL = confidence limit; SE = standard error; RR-resistance ratio; χ^2^ = chi-square value.

### The influence of diet with Precocene 1 on detoxification enzymes in *S. litura* larvae

**FIGURE 1:**
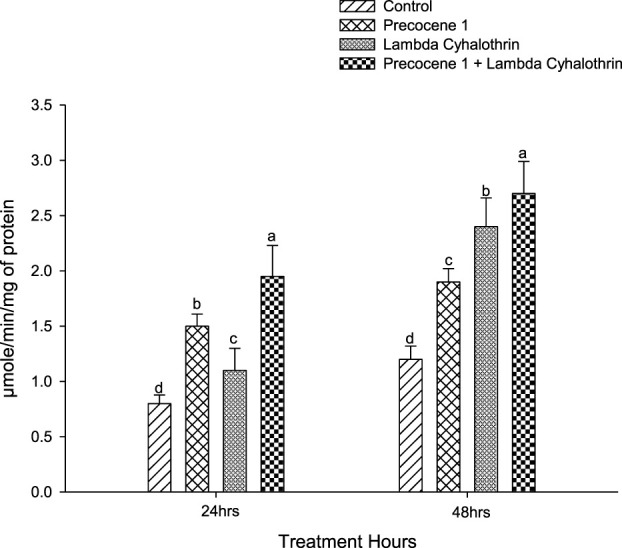
Effects of Precocene 1 on *Spodoptera litura* larvae tolerance to λ-cyhalothrin in α-esterase activity after 24 and 48 h. Data in the figure are means ± SE. Different letters above bars indicate significant differences (*p <* 0.05) according to the Tukey HSD test.

The activity of the α-esterase enzyme in the control, the effect of Precocene 1 only, of λ-cyhalothrin, and of the synergistic effect of Precocene 1 with λ-cyhalothrin in the larva of *S. litura* at 24 and 48 h after exposure is reported in Figure 1. The higher enzyme activity of *S. litura*, due to the various treatments, is shown in the order of Precocene 1 + λ-cyhalothrin > Precocene 1>λ-cyhalothrin > control and was 1.95, 1.5, 1.1, and 0.8, respectively, in the said order at 24 h of exposure. At 48 h of exposure, profound activity was observed in Precocene 1 + λ**-**cyhalothrin (2.7), followed by λ-cyhalothrin, Precocene 1, and the control (2.4, 1.9, and 1.2).

**FIGURE 2:**
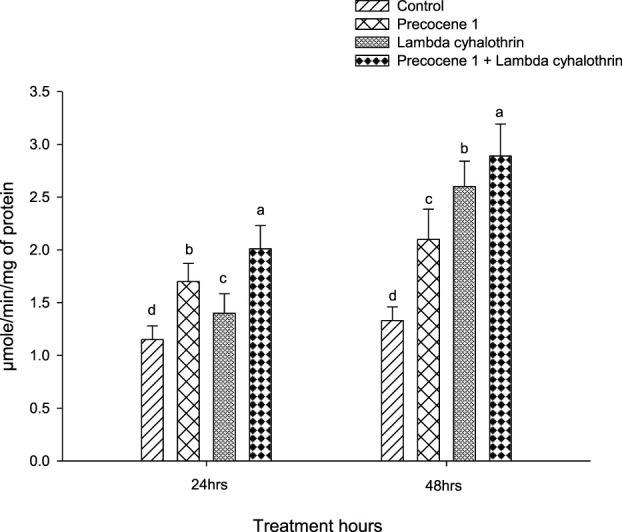
Effects of Precocene 1 on *Spodoptera litura* larvae tolerance to λ-cyhalothrin in β-esterase activity after 24 and 48 h. Data in the figure are means ± SE. Different letters above bars indicate significant differences (*p <* 0.05) according to the Tukey HSD test.

The β-esterase enzyme activity in the larvae of *S. litura* at 24 and 48 h of exposure in the control, Precocene 1, λ-cyhalothrin, and the combined effect of Precocene 1 + λ-cyhalothrin is shown in Figure 2. A similar trend in the enzyme activity in *S. litura* as in the case of alpha esterase was observed, and the values were 2.01, 1.7, 1.4, and 1.15 at 24 h of exposure. Similarly, in the larva of *S. litura*, the enzyme activity at 48 h of exposure was also found to be of the same order as reported in the alpha esterase activity in *S. litura* at 48 h, with values of 2.89, 2.6, 2.10, and 1.33.

**FIGURE 3:**
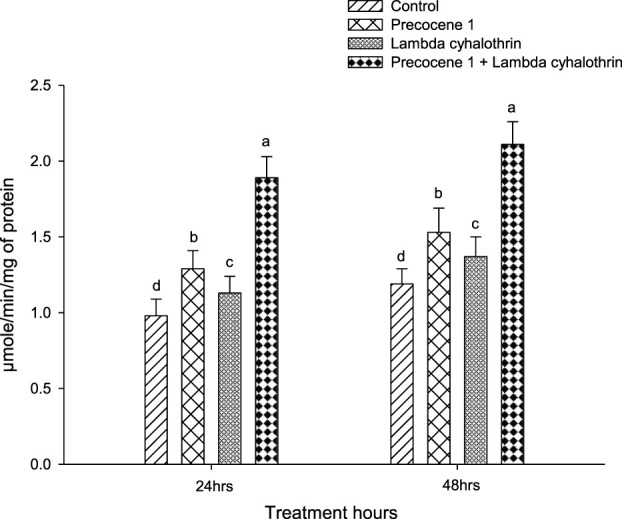
Effects of Precocene 1 on *Spodoptera litura* larvae tolerance to λ-cyhalothrin in glutathione-S-transferase activity after 24 and 48 h. Data in the figure are means ± SE. Different letters above bars indicate significant differences (*p <* 0.05) according to the Tukey HSD test.

Regarding GST enzyme activity, both at 24 and 48 h of exposure to the synergistic effect of Precocene 1 + λ**-**cyhalothrin, the activity of the enzymes was found to be greater, being 1.89 and 2.11 in the larva of *S. litura*. The enzyme activity of *S. litura* was followed in the order of Precocene 1, λ-cyhalothrin, and the control as 1.29, 1.13, and 0.98 at 24 h of exposure, while a similar rate of enzyme activity was seen in *S. litura* larva at 48 h of exposure (Figure 3).

**FIGURE 4:**
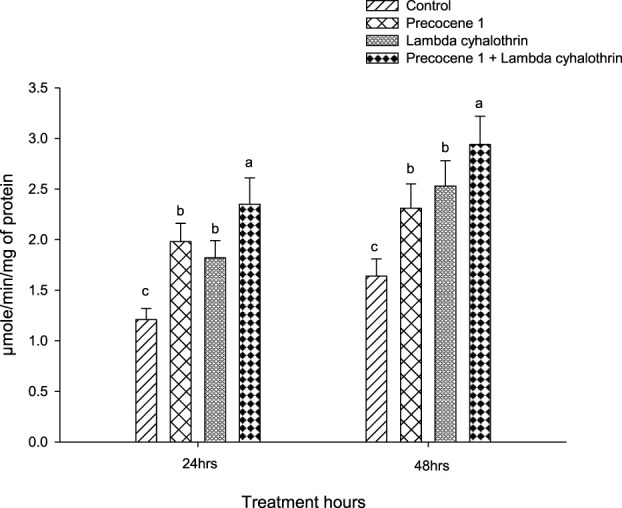
Effects of Precocene 1 on *Spodoptera litura* larvae tolerance to λ-cyhalothrin in cytochrome P450 activity after 24 and 48 h. Data in the figure are means ± SE. Different letters above bars indicate significant differences (*p <* 0.05) according to the Tukey HSD test.

The cytochrome P450 enzyme activity of *S. litura* in the control, Precocene 1, λ-cyhalothrin, and the synergistic effect of Precocene 1 + the λ-cyhalothrin treated group at 24 h was 1.21, 1.98, 1.82, and 2.35, whereas the values were 1.64, 2.31, 2.53, and 2.94 at 48 h of exposure, respectively, as shown in Figure 4.

### Expression responses of cytochrome P450 genes in *S. litura* on exposure to Precocene 1

**FIGURE 5:**
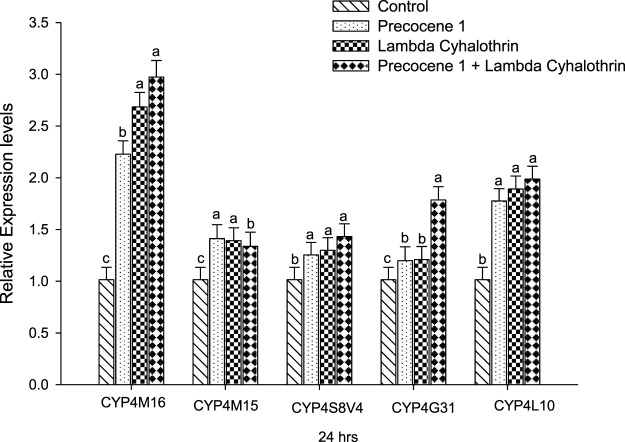
Effect of Precocene 1 on *Spodoptera litura* larvae tolerance to λ-cyhalothrin and relative expression levels of cytochrome P450s genes after 24 h. The transcription levels of three cytochrome P450s genes are determined by quantitative real-time PCR, normalized to different genes. Each bar indicates the mean of transcription levels (±SE), each being replicated. Different letters above bars indicate significant differences (*p* < 0.05) according to the Tukey HSD test.

The expressions of the genes *CYP4M16*, *CYP4M15*, *CYP4S8V4*, *CYP4G31*, and *CYP4L10* of *S. litura* at 24 h of exposure in the control, Precocene 1, λ-cyhalothrin, and Precocene 1 + λ-cyhalothrin groups are reported in Figure 5. Regarding *CYP4M16*, the gene expression of the larva was found to increase (1.01, 2.22, 2.68, and 2.97-fold). A similar increasing pattern of gene expression was noticed in *CYP4L10*, where the values were 1.01, 1.77 1.89, and 1.98-fold. When compared with the control in all other families, the gene expression was found to increase, whereas a changing pattern of gene expression was observed among the three groups. Among the five families studied, the *CYP4M16* showed higher gene expression due to the combined effect of Precocene 1 with λ-cyhalothrin at 24 h of exposure.

**FIGURE 6:**
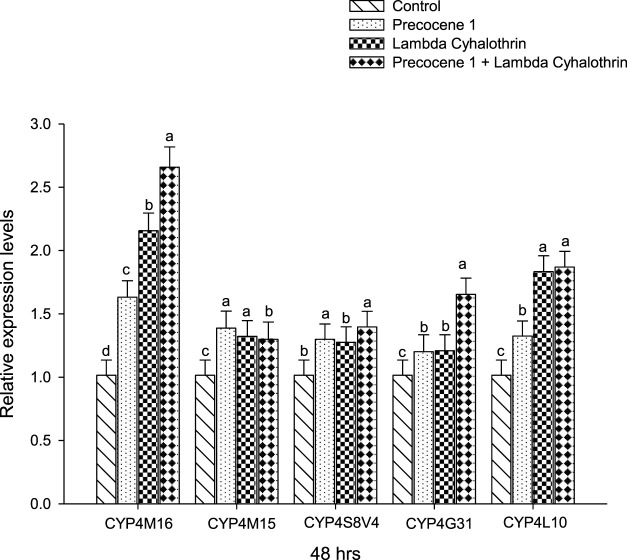
Effect of Precocene 1 on *Spodoptera litura* larvae tolerance to λ-cyhalothrin and relative expression levels of cytochrome P450s genes after 48 h. The transcription levels of three cytochrome P450s genes are determined by quantitative real-time PCR, normalized to different genes. Each bar indicates the mean of transcription levels (±SE), each being replicated. Different letters above bars indicate significant differences (*p* < 0.05) according to the Tukey HSD test.

The *CYP4M16*, *CYP4M15*, *CYP4S8V4*, *CYP4G31*, and *CYP4L10* expressions of *S. litura* after an exposure period of 48 h in the control, Precocene 1, λ-cyhalothrin, and Precocene 1 + λ-cyhalothrin groups are reported in Figure 6. A high level of gene expression was found in *CYP4M16*, with Precocene 1 + λ**-**cyhalothrin (2.65); the lowest level of gene expression (1.20) was observed in the Precocene 1-treated *CYP4G31* of *S. litura*; and no variation in the gene expression pattern was noticed in the control.

## Discussion

Plants produce a variety of secondary metabolites, or allelochemicals, which play a defensive role against plant eaters and pathogens. Furthermore, the natural predators of herbivores are attracted by these allelochemicals (Takabayashi et al., 1991; War et al., 2011). In the meanwhile, such feeding behavior paves the way to persistent development of resistance against pesticides in the agricultural field (Li et al., 2007; Zhu et al., 2016). The success of phytophagous insects is affected by the way they modulate their defensive strategies against the changing biotic stress of various kinds of secondary metabolites present in the plant. They do this by using the detoxification enzyme to detoxify or eliminate harmful components (Hafeez et al., 2018) Cytochrome P450 monooxygenases (P450s), esterases, and glutathione S-transferases (GST), are the prime detoxification enzymes to disarm insecticides and phytotoxins (Scott and Wen, 2001; Feyereisen, 2005; Li et al., 2007; Schuler, 2012; Liu et al., 2013). The present study investigated the effect of a diet incorporating Precocene 1 on the tolerance of *S*. *litura* larvae in response to λ-cyhalothrin. Furthermore, the impact of Precocene 1 on the activity of P450, esterases, and glutathione S-transferases, and the relative gene expression levels of cytochrome genes (*CYP4M16*, *CYP4M15, CYP4S8V4*, *CYP4G31*, and *CYP4L10*) were also assessed.

Concerning the synergistic ratio, findings on the individual effect of Precocene1 in an enhanced state, as opposed to the synergistic and lone effect of PBO, deviate from the results of Chen et al. (2018), while at the same time they coincide well with the findings of Hafeez et al. (2020). Adoption of the molecular strategy of upregulating P450 genes may be the mechanism behind the resistance (Elzaki et al., 2015). The enhanced α- and β-esterase activity at 24 and 48 h of exposure to the various treatments in the present study supports the results of other researchers (Mukherjee, 2003; Usha Rani and Pratyusha, 2013; Karthi and Shivakumar, 2016). Generally, the allelochemicals, be they either the secondary metabolites of the plant or the synthetic pesticides, developed toxicity when exposed to the pest, and in response, the pest nullified the same. The pest exhibited enhanced detoxifying enzyme activity, which may be the reason for the results of the present study. The increased levels of the detoxifying enzyme GST found in this study are in accordance with the reports of Dhivya et al. (2018), and Manjula et al. (2020), who identify the crucial role the multifunctional enzyme GST has in metabolizing the treatment of toxic plant allelochemicals, namely, hexane extract of *Prosopis juliflora*, and petroleum benzene leaf extract of *Manihot esculenta*, in *S. litura.* In the present study, the diet incorporating Precocene 1 led to an increased level of tolerance to the insecticide, λ-cyhalothrin, in *S. litura*. A similar kind of resistance to λ-cyhalothrin was noticed in *S. exigua* (Hafeez et al., 2020) and *H. armigera* (Chen et al., 2018), due to quercetin ingestion, and in *H*. *zea* for*α*-cypermethrin when exposed to xanthotoxin (Li et al., 2000). Likewise, tolerance to deltamethrin was observed in *S. exigua* fed with gossypol (Hafeez et al., 2018). The enhanced action of the P450 enzyme from 24 to 48 h, noticed in Precocene 1, λ-cyhalothrin, and Precocene 1 + λ-cyhalothrin, fed to larvae of S. *litura*, is corroborated by the results of Chen et al. (2018) and Tao et al. (2012) concerning *H. armigera* when fed with gossypol on exposure to pyrethroid, and quercetin to λ-cyhalothrin, respectively. In the result of RT-PCR at 24 and 48 h, the transcriptional levels of the CYP4M16, CYP4M15, CYP4S8V4, CYP4G31, and CYP4L10 enzymes of *S. litura* increased more markedly in all treatments other than in the control, and such activity resulted in the enhancement of P450 gene expression. The results of the present study accord with those of Li X. C. et al. (2004); Liu et al. (2006); and Rupasinghe et al. (2007), across a broad spectrum of compounds such as xanthotoxin, quercetin, and rutin, as well as the synthetic insecticides cypermethrin, diazinon, and aldrin.

## Conclusion

The impact of active compounds isolated from a natural plant could elevate sensitivity to insecticides by enhancing the activity of detoxification enzymes in agricultural insects. Furthermore, the cytochrome P450 enzyme system certainly plays a vital role in ways insects resist the plant’s chemical defense mechanisms. The current investigation assessed the impact of Precocene 1 alone, PBO, and their combined effect on the synergistic potential of *S. litura*, the role of plant allelochemicals in the activity of the detoxification enzymes *viz.*, esterase, GST, and Cytochrome P450, and also the gene expression levels of *CYP4M16*, *CYP4M15*, *CYP4S8V4*, *CYP4G31*, and *CYP4L10* in response to λ-cyhalothrin. It is obvious from the results that the *S. litura* showed different degrees of resistance, and after Precocene 1 treatment, particularly, the P450 gene showed a low level of expression, as in the control. Research is needed to discover the candidate genes that respond specifically to the natural toxins of plants, and also the impact of insecticides against such pests, in order to improve pest management strategies.

## Data Availability

The raw data supporting the conclusion of this article will be made available by the authors, without undue reservation.
