# Supplementary material for: Effects of phytocompound Precocene 1 on the expression and functionality of the P450 gene in λ-cyhalothrin-resistant Spodoptera litura (Fab.)
Source: Front Physiol. 2022 Nov 10;13:900570. doi: 10.3389/fphys.2022.900570 (PMC9684723; doi:10.3389/fphys.2022.900570)
Supplement: Supplementary file 1 [file DataSheet1.docx]

Table 1. Primers used in this study for quantitative real time polymerase chain reaction (qRT-PCR).

| S. No | Primer Name | Primer Sequence (5'-3') | Size (bp) | NCBI Accession Numbers |
| --- | --- | --- | --- | --- |
| 1. | CYP4M16 | GGCGAACGAACCTGAAATA  CTTCATCTGACTCAAGTCTTCC | 103 | DQ355382 |
| 2. | CYP4M15 | CCCACCAGTGCACTTTATTA  GCAGGTCTAAGATCAGAATGT | 104 | DQ352139 |
| 3. | CYP4S8v4 | AGTATTTGGAGGCAGTCATC  CGTACCCTTCTTCACTGTTAT | 116 | DQ355381 |
| 4. | CYP4G31 | CACCCTGCAGATGAAGTATT  CGTAGTTGTTGGTAGCGATT | 119 | DQ350813 |
| 5. | CYP4L10 | TTGAGCGAAGGATAACAAGAG  CTGGTTGGCGTTGAATCT | 99 | DQ352134 |

Table 2. Synergistic effect of piperonyl butoxide on the susceptibility of third-instar larvae of *S. litura* to lambda-cyhalothrin after the ingestion of Precocene 1.

| Treatment | LC_50_ (mg a.i./L) | 95 % CL | Slope ± SE | df | ᵪ^2^ | P-value |
| --- | --- | --- | --- | --- | --- | --- |
| Piperonyl Butoxide (PBO) | 91.89 | 81.67 ± 99.08 | 3.154 ± 0.39 | 3 | 2.157 | *P<0.01* |
| Precocene 1 | 78.05 | 71.08 ± 86.05 | 2.871 ± 0.67 | 3 | 1.763 | *P<0.01* |
| Precocene 1 + Piperonyl Butoxide (PBO) | 61.06 | 55.04 ± 68.87 | 2.172 ± 0.05 | 3 | 1.235 | *P<0.01* |

Where, LC_50_ = lethal concentration to kill 50% of the population; a.i. = active ingredient; CL = confidence limits; SE = standard error; df = degree of freedom; **ᵪ**^2^ = Chi-square value.
